# RETRACTION: Effects of Non‐Pharmacological Interventions on Pain Intensity of Children With Burns: A Systematic Review and Meta‐Analysis

**DOI:** 10.1111/iwj.70239

**Published:** 2025-02-25

## Abstract

**RETRACTION:** FarzanR. , ParviziA. , HaddadiS. , TabarianM. S. , JamshidbeigiA. , SamidoustP. , VajargahP. G. , MollaeiA. , TakasiP. , KarkhahS. , FiroozM. , and HosseiniS. J. , “Effects of Non‐Pharmacological Interventions on Pain Intensity of Children With Burns: A Systematic Review and Meta‐Analysis,” International Wound Journal 20, no. 7 (2023): 2898–2913, 10.1111/iwj.14134.36859758 PMC10410361

The above article, published online on 01 March 2023, in Wiley Online Library (http://onlinelibrary.wiley.com/), has been retracted by agreement between the journal Editor in Chief, Professor Keith Harding; and John Wiley & Sons Ltd. A third party reported to the journal that they had found evidence of excessive self‐citations in the reference list of this article. The publisher confirmed multiple instances of redundant self‐citations. Upon further investigation, the publisher also concluded that this article was accepted solely on the basis of a compromised peer review process. In view of the clear evidence of compromised peer review, the parties agreed that the paper must be retracted. The authors disagree with the retraction.

**RETRACTION:** R. Farzan , A. Parvizi , S. Haddadi , M. S. Tabarian , A. Jamshidbeigi , P. Samidoust , P. G. Vajargah , A. Mollaei , P. Takasi , S. Karkhah , M. Firooz , and S. J. Hosseini , “Effects of Non‐Pharmacological Interventions on Pain Intensity of Children With Burns: A Systematic Review and Meta‐Analysis,” International Wound Journal 20, no. 7 (2023): 2898–2913, 10.1111/iwj.14134.36859758 PMC10410361

The above article, published online on 01 March 2023, in Wiley Online Library (http://onlinelibrary.wiley.com/), has been retracted by agreement between the journal Editor in Chief, Professor Keith Harding; and John Wiley & Sons Ltd. A third party reported to the journal that they had found evidence of excessive self‐citations in the reference list of this article. The publisher confirmed multiple instances of redundant self‐citations. Upon further investigation, the publisher also concluded that this article was accepted solely on the basis of a compromised peer review process. In view of the clear evidence of compromised peer review, the parties agreed that the paper must be retracted. The authors disagree with the retraction.

